# High Aluminum Drives Different Rhizobacterial Communities Between Aluminum-Tolerant and Aluminum-Sensitive Wild Soybean

**DOI:** 10.3389/fmicb.2020.01996

**Published:** 2020-08-19

**Authors:** Qihan Shi, Jing Jin, Yuantai Liu, Yafeng Zhang, Zhandong Cai, Qibin Ma, Yanbo Cheng, Ronghui Wen, Hai Nian, Tengxiang Lian

**Affiliations:** ^1^The State Key Laboratory for Conservation and Utilization of Subtropical Agro-bioresources, South China Agricultural University, Guangzhou, China; ^2^The Key Laboratory of Plant Molecular Breeding of Guangdong Province, College of Agriculture, South China Agricultural University, Guangzhou, China; ^3^The State Key Laboratory for Conservation and Utilization of Subtropical Agro-bioresources, College of Life Science and Technology, Guangxi University, Nanning, China

**Keywords:** aluminum toxicity, wild soybean, 16S rRNA high-throughput sequencing, bacterial communities, co-occurrence network

## Abstract

Aluminum (Al)-resistant plant cultivars can recruit beneficial microbes to alleviate the stresses. However, the mechanism of how rhizobacterial communities strengthen Al tolerance of wild soybean has not been addressed. The aim of this study was to investigate the bacterial community structure in the rhizosphere of Al-tolerant (BW69) and Al-sensitive (W270) wild soybean germplasm subjected to three Al concentrations. We analyzed the rhizobacterial communities of the two genotypes by high-throughput sequencing of 16S rRNA genes. The results showed that high Al stress recruited different rhizobacterial communities between two genotypes. In total, 49 OTUs, such as OTU15 (*Gammaproteobacteria_KF-JG30-C25_norank*), OTU23 (*Mizugakiibacter*), and OTU93 (*Alkanibacter*), were enriched in the rhizosphere of BW69 at the low and high Al concentrations. Moreover, bacterial community in the rhizosphere of BW69 had a more complex co-occurrence network than did W270 at the high Al concentration. Overall, our findings highlighted that high Al concentration magnified the difference in rhizobacterial community structure between two genotypes. However, the lower modularity of the co-occurrence network in rhizosphere of BW69 than W270 under Al stress may cause the rhizobacterial community to be less resistant and more influenced by disturbance. This study emphasizes the possibility of using rhizobacteria as an improved crop breeding or gene to produce crops that are more resistant to the toxicity of heavy metal.

## Introduction

Acidic soils with pH less than 5.5 account for about 30% of the world’s arable soils (von Uexküll and Mutert, 1995). Aluminum (Al) toxicity is one of the crucial factors limiting the growth of crops in acidic soils (Kochian et al., 2005). The main toxicity of Al to plants inhibits root growth and the absorption of water and nutrients (Liang et al., 2001). For instance, Al^3+^ could bind with phosphorus (P) in the soil and lead to the P deficiency (Verma et al., 2005; Zelinová et al., 2009). Al also reduces the conversion efficiency of NO_3_^–^ to NH_4_^+^ and thus further increases the toxicity of Al because NH_4_^+^-N could mitigate the toxicity of Al (Matsumoto and Yamaya, 1986; Rengel and Elliott, 1992; Ma et al., 2002; Zhao and Shen, 2018).

The secretion of organic acids from roots, such as citric acid, oxalic acid, and malic acid, is one of the important mechanisms for plant to alleviate Al toxicity (Ma et al., 2001). These organic acids released from plant roots can chelate Al ions, reducing the toxicity of Al to plants (Ma et al., 2001; Kochian et al., 2005). The type and quantity of organic acids vary among different genotypes (Miyasaka et al., 1991). In wheat, maize, and soybean, Al-tolerant (Al-T) varieties secrete much more organic acids than Al-sensitive (Al-S) varieties (Pellet et al., 1995; Ryan et al., 1995; Yang et al., 2001). Furthermore, the remarkable differences in organic acids between Al-T and Al-S varieties directly affect the microbial community structure, abundance, and diversity in the rhizosphere (Germida and Siciliano, 2001; Yang et al., 2012; Peiffer et al., 2013). The root exudates secreted from stress-resistant genotypes can recruit beneficial microbes to alleviate the stresses, via regulating microbial metabolic activities and changing nutrient availability in soil (Fierer and Jackson, 2006; Parniske, 2008; West et al., 2010; Shi et al., 2011; Bulgarelli et al., 2013; Li et al., 2014; Kwak et al., 2018). For instance, some secondary metabolites from root exudates likely attract some arbuscular mycorrhizal fungi and rhizobia, which may produce compounds and in turn increase the secretion of organic acids to alleviate Al toxicity (Pellet et al., 1995; Phillips et al., 2004; Besserer et al., 2006; Berendsen et al., 2012; Yang et al., 2012). Moreover, some Al-tolerant bacteria (e.g., *Klebsiella* and *Serratia*) in soil could form Al^3+^–siderophore complexes and promote P uptake to alleviate Al phytotoxicity (Mora et al., 2017). In addition, exudates such as organic acids, protons, and acid phosphatases produced by the microbes can also reduce the impact of Al toxicity on plants or increase the available phosphorus in the rhizosphere, indirectly help plants reduce aluminum toxicity and adapt to acidic soils (Cabral et al., 2015).

Wild soybean (*Glycine soja* L.) is a close ancestor of cultivated soybean (*Glycine max* L.) (Hymowitz, 1970). Compared with cultivated soybean, wild soybean has stronger resistance to biotic and abiotic stresses, such as drought, cold, Al, and attack by viruses and insects (Dong et al., 2001; Chen et al., 2006; Singh and Hymowitz, 2011). In addition to the abundant resistance genes in wild soybeans, microorganisms in the rhizosphere also play an important role in resisting Al toxicity (Luo et al., 2013; Ma et al., 2018; Lian et al., 2019). Although many plant growth–promoting bacteria (PGPR) exert beneficial effects on various crops, their performance can be strongly cultivar specific (Montalban et al., 2017; Rodriguez et al., 2019). Previous research found that the composition and diversity of microorganisms in the rhizosphere of wild rice were significantly different from those in cultivated rice, and the enriched taxa affiliated to Anaerolineae and Nitrospirae in the rhizosphere of wild rice might benefit the disease resistance of the plants (Shenton et al., 2016). Moreover, our previous study reported that the impact of high Al stress on bacterial community structure differed in the rhizosphere between Al-T and Al-S cultivated soybeans, and Al-T soybean could recruit specific microorganisms to resist Al stress (Lian et al., 2019; Shi et al., 2020). However, the effect of wild soybean genotypes on the bacterial community in the rhizosphere has not been clarified, and the bacterial composition in the rhizosphere in response to different levels of Al stress was undefined either. Such studies may provide an important strategy for the identification of beneficial bacteria that may improve plants tolerance to Al stress. Therefore, this study could create a new window for research under acidic soil condition for explore the microbial resources of wild soybeans and crop production.

To investigate the mechanism of how rhizobacterial communities strengthen the Al tolerance of wild soybean, we analyzed the bacterial community structures in the rhizosphere of the Al-T (BW69) and Al-S (W270) genotypes of wide soybean, under different Al concentrations. The chemical properties of the rhizosphere were measured and their correlations with bacterial communities were examined. Given that BW69 is better adapted to Al stress than W270, we hypothesized that (1) there would be significant difference in bacterial structure between BW69 and W270, and (2) BW69 would recruit specific microorganisms in the rhizosphere that may play important roles in the resistance to Al stress.

## Materials and Methods

### Soil and Plant Materials

The soil used in the experiment was collected at Yingde County (113°40’N, 24°18’E), Guangdong Province, China. The soil was classified as Ali-Udic Argosol according to USDA soil taxonomy. The soil had a pH of 5.19, 10.1 g kg^–1^ total carbon, 0.39 g kg^–1^ total nitrogen, 19.40 g kg^–1^ total potassium, 1.27 g kg^–1^ total phosphorus, 60.4 mg kg^–1^ available phosphorus, 40.1 mg kg^–1^ ammonium nitrogen (NH_4_^+^-N), 12.6 mg kg^–1^ nitrate nitrogen (NO_3_^–^-N), 0.01 cmol kg^–1^ exchangeable H^+^, and 0.15 cmol kg^–1^ exchangeable Al^3+^. In this study, soybeans used in the experiment include Al-T (BW69) and Al-S (W270), two wild genotypes (Ma et al., 2018).

### Experimental Design and Soil Sampling

A pot experiment was carried out in a greenhouse at South China Agricultural University in Guangzhou, China. In this study, there were three concentrations of Al in the form of pelletized Al_2_(SO_4_)_3_^⋅^18H_2_O, i.e., (1) 0 Al^3+^ g kg^–1^, (2) 0.2 Al^3+^ g kg^–1^, and (3) 0.4 Al^3+^ g kg^–1^, representing zero, low, and high Al concentrations, respectively. A no-plant control (CK) was set at each Al concentration. There were five replicates for each treatment. Each pot was filled with 1.5 kg of soil (sieved with 4 mm mesh) and four seeds with uniform size were sown into each pot on June 15, 2018. The pots were placed in a greenhouse with a temperature range of 16–20°C at night and 28–32°C during the daytime. The soil moisture content was controlled at 80% of the field water capacity by weighing and watering. The loose attached soil was removed by shaking the roots at the flowering stage (60 days after sowing), and then the roots were washed with phosphate-buffered saline (Shi et al., 2015). A total of 45 rhizosphere soil samples were collected. From each sample, 2 g was placed into a micro-centrifuge tube and kept at -80°C until DNA extraction. The remaining rhizosphere soil was kept at 4°C before the determination of soil characteristics. Meanwhile, the wild soybean plants were harvested and dried 70°C for 3 days (Lian et al., 2017).

### Determination of Soil Properties

Soil pH was determined in a 5:1 water-to-soil suspension using a pH meter (FE20-FiveEasy pH, Germany) (Wang et al., 2013). Total carbon and total nitrogen were determined with an elemental analyzer (VarioEL III, Germany) (Jones and Willett, 2006). Soil total potassium was measured by inductively coupled plasma-atomic emission spectrometry (I-7500; Shimadzu, Japan) (Jiao et al., 2019). The soil-exchangeable Al^3+^ and H^+^ were measured by a titrimetric method (Abreu et al., 2003). Soil NO_3_^–^-N and NH_4_^+^-N levels were determined with a continuous flow chemistry analyzer (skalar SAN++, Netherlands) (Miranda et al., 2001). Available phosphorus and total potassium were determined using an atomic absorption spectrometer (GFA-6800, Japan) (Sun et al., 2015).

### Extraction of Soil DNA and Real-Time Quantitative PCR

A Fast DNA SPIN Kit for Soil (MP Biomedicals, Santa Ana, CA) was used to extract DNA following the manufacturer’s specifications. The bacterial community abundance was determined by quantitative real-time PCR (q-PCR) (ABI 7900) using the primers 515f (5′-GTGCCAGCMGCCGCGGTAA-3′) and 907r (5′-CCGTCAATTCMTTTRAGTTT-3′) (Osburn et al., 2011). The 20 μl PCR mixture included 1.0 μl of extracted DNA, 1.0 μl forward and reverse primers (10 nM), 7.0 μl of sterile water, and 10 μl SYBR Premix Ex Taq (Takara, Dalian, China). The q-PCR amplification program was as follows: initial denaturation for 1 min at 98°C, followed by 30 cycles of denaturation for 10 s at 98°C, annealing for 30 s at 50°C, elongation for 60 s at 72°C, and the final cooling for 5 min at 72°C to perform a cycle. The copy number of 16S rRNA gene was calculated based on standard curves (Yao et al., 2014).

### High-Throughput Sequencing and Data Analysis

Based on Illumina MiSeq sequencing, the 515F/907R primer sequence was used to amplify the V4 hypervariable region of the 16S rRNA gene (Osburn et al., 2011). Equimolar amounts of amplicons from the same sample were pooled, then the Illumina paired-end (PE 250) sequencing platform was used for double-end sequencing analysis. All raw sequences are available in the NCBI short-read archive under accession number PRJNA531335.

After sequencing, the FASTQ original sequence files of bacteria were processed by USEARCH 10 (Edgar, 2013). The unoise3 algorithm was conducted for denoising (error-correction) amplicon reads. Briefly, reads with sequencing errors were detected and corrected, and chimeras were identified and removed using the Uchime algorithm (Edgar et al., 2011). To obtain the corresponding species classification information for each operational taxonomic unit (OTU), a classification analysis of OTU representative sequences with 97% similarity was conducted using the RDP classifier with a Bayesian algorithm (Cole et al., 2009). Chao1 richness and Shannon index were calculated using Mothur V1.30.1 (Kwak et al., 2018).

Genstat V12 was used to perform a one-way ANOVA to identify significant differences in soil chemical properties among all treatments (Lian et al., 2016). Non-metric multi-dimensional scaling (NMDS), canonical correspondence analysis (CCA), Adonis test, and Mantel test were conducted using R v.3.5.1 with the “vegan” package (R Development Core Team, 2006). To identify the OTUs that were significantly different between two genotypes, a generalized linear model with a negative binomial distribution of the OTU relative abundance was created to normalize the values of each OTU (Edwards et al., 2015). A Venn analysis was conducted to show the co-enriched OTUs according to the results of the generalized linear model (Edwards et al., 2015). Based on the 95% confidence interval, significantly different genera between BW69 and W270 were analyzed by Stamp v.2.1.3 (Parks et al., 2014).

### Co-occurrence Network Analysis of Bacterial Community

We constructed six networks to analyze pairwise correlations of bacterial OTUs (average abundance > 0.1%). Spearman’s rank correlation and *p*-values were calculated in the “psych” R package, using the Gephi for visualization (Jiang et al., 2017a). A correlation was considered as significance with Spearman’s correlation coefficient > 0.8 and *p* < 0.05 (Jiang et al., 2017b). The nodes in the co-occurrence network represent bacteria OTUs, and edges represent robust and significant correlations between OTUs. A set of network topological properties (e.g., average degree, average path length, and graph density) and node features (e.g., degree, closeness centrality, and betweenness centrality) were calculated in Gephi. Nodes with high degree, high closeness centrality, and high betweenness centrality values are considered as keystone species (Zhou et al., 2010; Berry and Widder, 2014; Agler et al., 2016).

## Results

### Soybean Biomass, Bacterial Abundance, and Soil Chemical Properties

**FIGURE 1:**
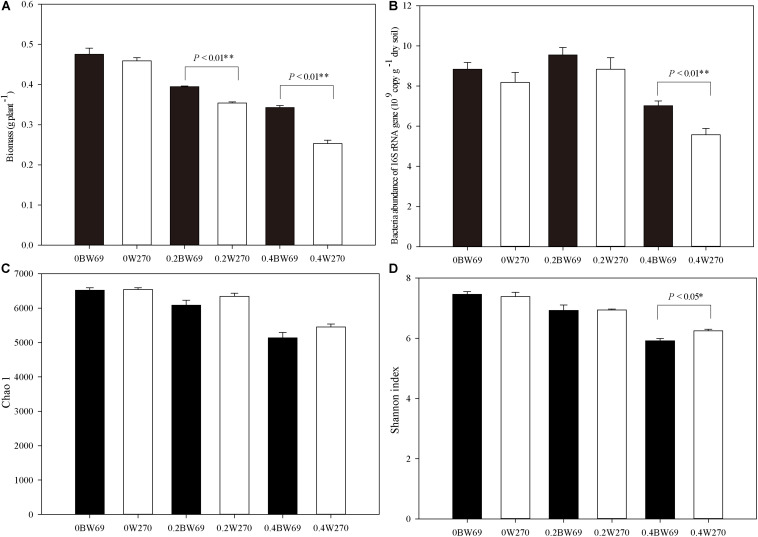
Effects of Al addition (0, 0.2, and 0.4 g kg^–1^) on the biomass **(A)**, abundance of bacterial 16S rRNA gene copies **(B)**, Chao1 index **(C)**, and Shannon index in the rhizosphere of wild soybean **(D)**. One-way ANOVA with Student’s *t*-test showed significant differences between BW69 and W270 (*p* < 0.05). Error bars on data points represent the SEM (*n* = 5).

Overall, Al stress decreased the biomass of both soybean genotypes, and the biomass of BW69 was significantly higher than W270 at the low and high Al concentration (Figure 1A). The bacterial abundance ranged from 4.3 × 10^9^ to 10.9 × 10^9^ gene copies g^–1^ dry soil across all samples. A significant difference in bacterial abundance between BW69 and W270 was observed at the high Al concentration only (*p* < 0.01) (Figure 1B).

The effect of Al stress on soil chemical properties was summarized in Table 1. Generally, soil available phosphorus, NH_4_^+^-N and NO_3_^–^-N were significantly higher in the rhizosphere of BW69 than W270 at the low and high Al concentrations. However, the concentrations of soil total potassium, exchangeable Al^3+^, and pH were significantly different between two genotypes only at the high Al concentration, with higher values for W270. Moreover, the soil-exchangeable H^+^ and Al^3+^ concentrations increased with the increase of Al concentration supplied, whereas pH showed the opposite trend (Table 1).

**TABLE 1:** Effects of different wild soybean cultivars and Al concentrations on soil chemical properties in an acidic soil.

Cultivars	Al concentration (g kg^–1^)	TC g kg^–1^	TN g kg^–1^	C:N	TK g kg^–1^	TP g kg^–1^	AP mg kg^–1^	NH_4_^+^-N mg kg^–1^	NO_3_^—^N mg kg^–1^	Exchangeable H^+^ cmol kg^–1^	Exchangeable Al^3+^ cmol kg^–1^	pH
CK	0	10.11a	0.39a	26.18d	19.40ab	1.27a	60.39b	40.12bcd	12.60b	0.01d	0.15d	5.19b
BW69	0	10.09a	0.39a	25.57d	18.14abc	1.26ab	59.41b	69.84a	17.00a	0.04d	0.06d	5.32a
W270	0	9.72ab	0.34bc	29.02bcd	17.64abc	1.30a	69.44a	78.90a	17.00a	0.03d	0.05d	5.29a
CK	0.2	8.91c	0.36ab	24.66d	14.92de	1.21c	35.45cd	38.50bcd	9.01c	0.22c	0.51c	4.17c
BW69	0.2	10.16a	0.32bc	32.04ab	17.17bcd	1.26a	53.43b	77.98a	14.87ab	0.28bc	0.64c	4.16c
W270	0.2	9.14bc	0.29cd	31.37abc	16.87cde	1.14bc	41.52c	51.70b	9.04c	0.28bc	0.68c	4.19c
CK	0.4	8.13d	0.29cd	28.24bcd	14.64e	1.08c	26.26e	37.51cd	2.81d	0.39a	2.79a	3.80d
BW69	0.4	8.62cd	0.32bc	26.93cd	17.18bcd	1.14c	38.10c	50.33bc	6.64c	0.38ab	2.40b	3.67e
W270	0.4	8.89c	0.25d	35.10a	19.56a	1.10c	29.10de	34.31d	3.14d	0.47a	3.05a	3.78d

**Significant differences were indicated with different lowercase letters (p < 0.05, one-way ANOVA, n = 5). pH, soil pH; TC, total carbon; TN, total nitrogen; C:N, ratio of TC to TN; TP, total phosphorus; AP, available phosphorus; NH_4_^+^-N, ammonium nitrogen; NO_3_^–^-N, nitrate nitrogen.**

### Taxonomic Classification and Soil Bacterial Diversity

A total of 2,071,056 high-quality sequences were obtained in this study, and the average reading count for each sample was 28,309. When clustered with 97% sequence identity, there were 8108 OTUs for all samples, with a mean of 4222 OTU for each sample. The Chao1 and Shannon index of the bacterial community in the rhizosphere of two genotypes decreased with the increase of Al concentration. However, Shannon index only showed a significant difference between two genotypes at the high Al concentration, with the higher value for W270 (Figures 1C,D).

### Structure of the Soybean Rhizobacterial Community

**FIGURE 2:**
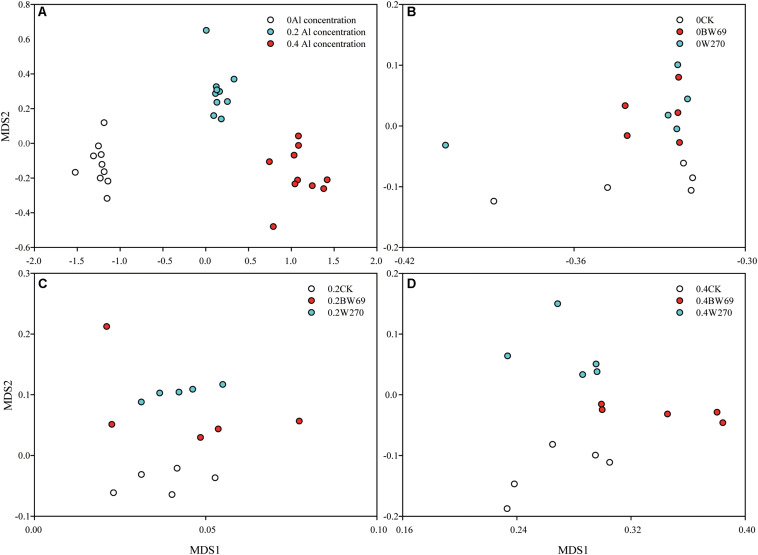
Non-metric multidimensional scale (NMDS) analysis based on Bray–Curtis dissimilarity showing that the rhizobacterial communities under 0, 0.2, and 0.4 g kg^–1^ of Al concentrations separated from each other (Adonis test, *p* < 0.05) **(A)**. NMDS analysis based on Bray–Curtis dissimilarity that rhizobacterial communities of BW69 mixed those of W270 under the 0 g kg^–1^ Al concentration (Adonis test, *p* > 0.05) **(B)**. NMDS analysis based on Bray–Curtis dissimilarity that rhizobacterial communities of BW69 mixed with those of W270 under the 0.2 g kg^–1^ Al concentration (Adonis test, *p* > 0.05) **(C)**. NMDS analysis based on Bray–Curtis dissimilarity that rhizobacterial communities of BW69 separated from those of W270 under the 0.4 g kg^–1^ of Al concentration (Adonis test, *p* < 0.01) **(D)** (*n* = 5).

Based on the NMDS, the bacterial communities at three Al concentrations were significantly separated (Adonis test, *p* < 0.05) (Figure 2A). The rhizobacterial community for no-plant control samples significantly separated from BW69 and W270 at each Al concentration (Adonis test, *p* < 0.05) (Figure 2A). Significant differences between BW69 and W270 were only observed under the high Al concentration (Adonis test, *p* < 0.01) (Figures 2B–D). The bacterial community structure in the rhizosphere varied with Al concentration and genotype, and high Al concentration magnified the differences of bacterial community structure between two genotypes.

**FIGURE 3:**
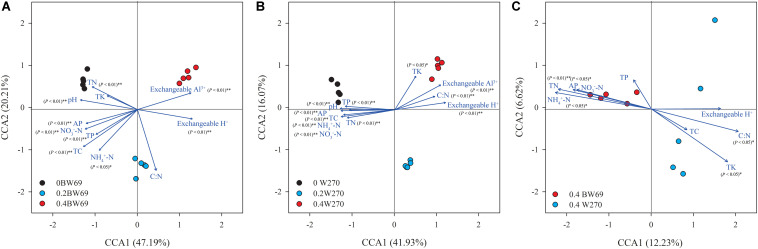
A canonical correspondence analysis (CCA) based on OTU level reveals the influence of soil properties on rhizobacterial communities under among BW69 samples **(A)**, W270 samples **(B)**, and high Al concentration samples **(C)**. Relationships between rhizosphere bacterial communities and soil properties. Arrows indicate the direction and magnitude of environmental parameters associated with bacterial community structure. pH, soil pH; TC, total carbon; TN, total nitrogen; C:N, ratio of TC to TN; TP, total phosphorus; AP, available phosphorus; NH_4_^+^-N, ammonium nitrogen; NO_3_^–^-N, nitrate nitrogen.

The CCA was used to determine the relationship between soil chemical properties and the bacterial communities in the rhizosphere. The CCA and Mantel test showed that the bacterial community structures were significantly related to the soil properties in rhizospheres of the two genotypes, which were total carbon, total nitrogen, total potassium, available phosphorus, NH_4_^+^-N, NO_3_^–^-N, exchangeable H^+^, exchangeable Al^3+^, and pH (Figures 3A,B and Supplementary Table S1). Moreover, the total nitrogen, C:N ratio, NH_4_^+^-N, available phosphorus, total potassium, and pH were significantly related to the bacterial community structure of the two genotypes at the high Al concentration (Figure 3C and Supplementary Table S1).

### The Relative Abundance of Soybean Rhizosphere Bacteria

**FIGURE 4:**
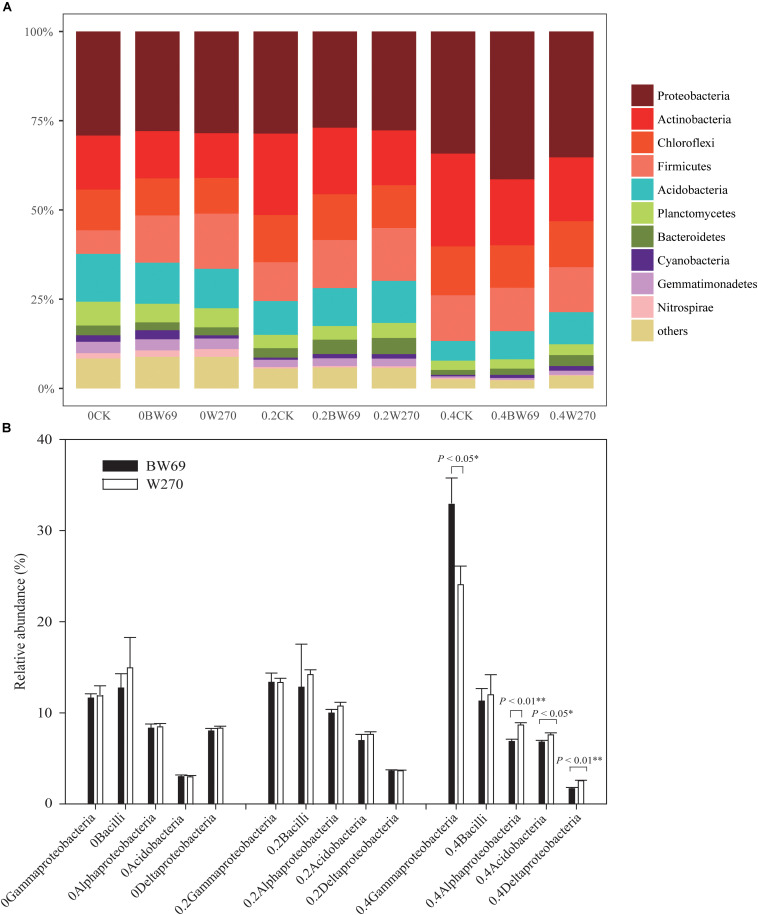
Relatively abundance of bacteria at the phylum level **(A)** and class level (top five dominant classes) **(B)** of the BW69 and W270 in 0, 0.2, and 0.4 g kg^–1^ Al concentration addition. One-way ANOVA with Student’s *t*-test showed significant differences between BW69 and W270 (*p* < 0.05). Error bars on data points represent the SEM (*n* = 5).

Proteobacteria, Actinobacteria, Chloroflexi, Firmicutes, and Acidobacteria were the dominant phyla in all soil samples, with total relative abundance over 75%; the relative abundance of the five phyla ranged from 21.4 to 47.7%, 11.1 to 35.6%, 47.7 to 11.1%, 4.8 to 31.8%, and 16.3 to 31.8%, respectively (Figure 4A and Supplementary Figure S1). Moreover, the phyla of Planctomycetes, Bacteroidetes, Cyanobacteria, Gemmatimonadetes, and Nitrospirae exhibited low abundances in all soil samples (relative abundance > 0.1%, but < 5%) (Figure 4A and Supplementary Figure S1). There was no significant difference between BW69 and W270 for the five dominant phyla. However, at the class level, the relative abundances of Alphaproteobacteria, Deltaproteobacteria, and Acidobacteria in the rhizosphere of W270 was higher than BW69 under the high Al concentration, whereas the Gammaproteobacteria were more abundant in BW69 (*p* < 0.05, *p* < 0.01) (Figure 4B).

**FIGURE 5:**
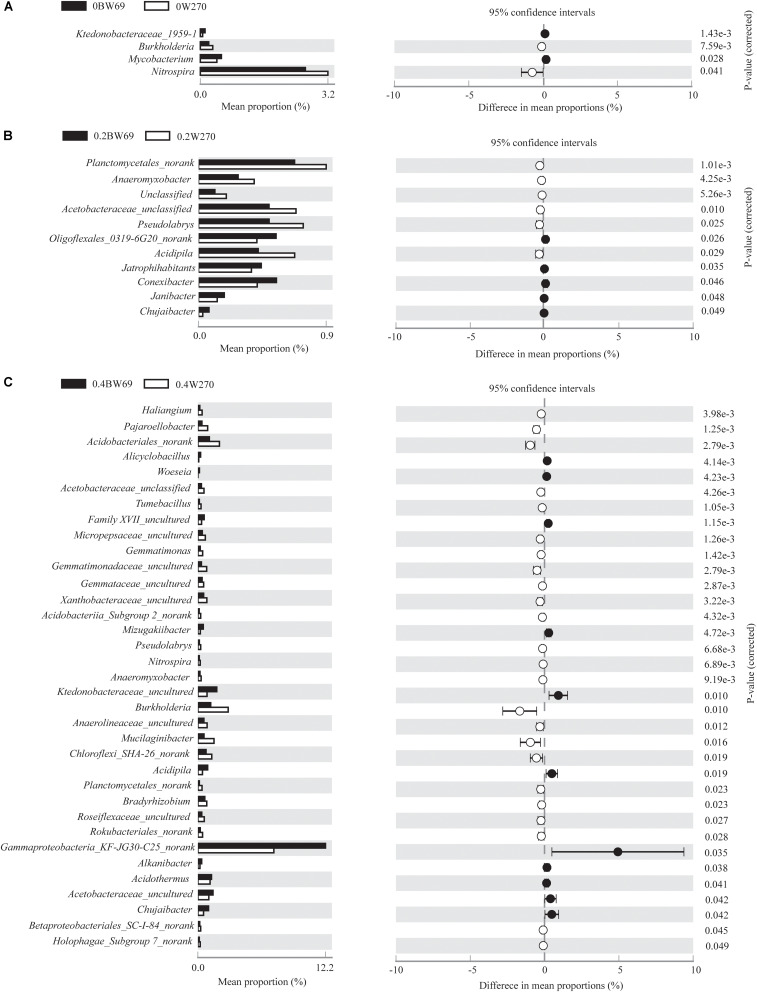
Stamp analysis of the relative abundance of genera between BW69 and W270 at the 95% confidence interval level under Al concentrations of 0 g kg^–1^ **(A)**, 0.2 g kg^–1^ **(B)**, and 0.4 g kg^–1^ **(C)**. *P*-values were calculated using Welch’s *t*-test.

Using the 95% confidence interval, the bacterial genera between BW69 and W270 at three Al concentrations were compared. The relative abundance of the top-100 genera were selected for further statistical analyses. The number of genera with significant differences between BW69 and W270 were 4, 11, and 35 at the zero, low, and high Al concentrations, respectively. Among these genera, two and five genera had higher relative abundance in the rhizosphere of BW69 compared with W270 at the zero and low Al concentrations, respectively (Figures 5A,B). Eleven genera, such as *Gammaproteobacteria_KF-JG30-C25_norank*, *Ktedonobacteraceae*_*uncultured*, and *Chujaibacter*, were significantly higher in BW69 compared with W270 at the high Al concentrations (Figure 5C).

**FIGURE 6:**
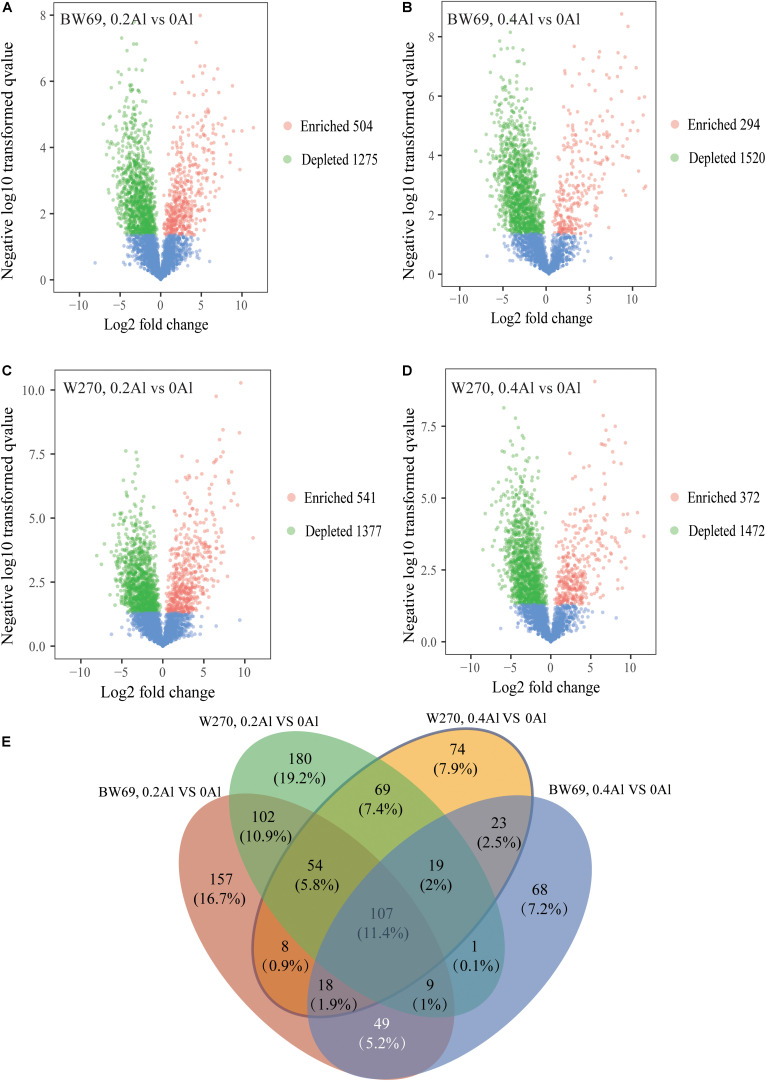
Enrichment and depletion of OTUs in 0.2 g kg^–1^ vs. 0 g kg^–1^ Al concentration of BW69 **(A)**, 0.4 g kg^–1^ vs. 0 g kg^–1^ Al concentration of BW69 **(B)**, 0.2 g kg^–1^ vs. 0 g kg^–1^ Al concentration of W270 **(C)** and 0.4 g kg^–1^ vs. 0 g kg^–1^ Al concentration of W270 **(D)**. Each point represents an individual OTU, and the position along the *y*-axis represents the abundance fold change compared with BW69 and W270, respectively, under the 0 g kg^–1^ Al concentration. The Venn diagram shows the number of unique enriched and co-enriched OTUs in the rhizospheres of BW69 and W270 under different Al concentrations **(E)**.

**FIGURE 7:**
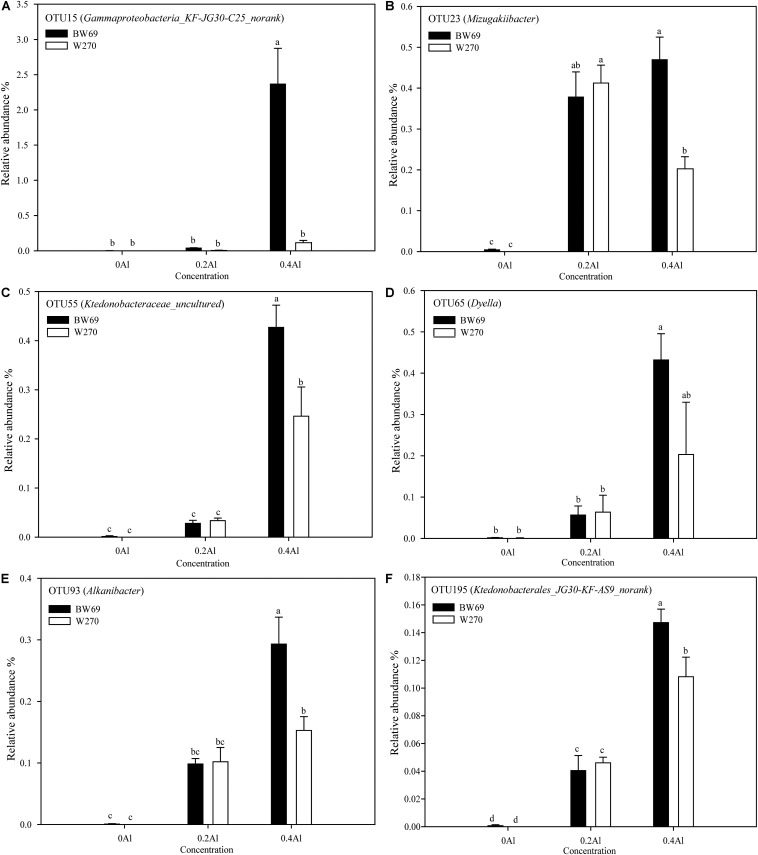
The relative abundance of dominant OTU15 **(A)**, OTU23 **(B)**, OTU55 **(C)**, OTU65 **(D)**, OTU93 **(E)**, and OTU195 **(F)**. Significant differences are indicated with different lowercase letters (*p* < 0.05, one-way ANOVA with Student’s *t*-test). Error bars on data points represent the standard error of the mean (*n* = 5).

To evaluate the difference of OTU between BW69 and W270 at different Al concentrations, we created a generalized linear model of the negative binomial distribution. OTU counts in the rhizospheres of BW69 and W270 under the zero Al concentration were used as the controls to compare the enriched or depleted OTUs under the low and high Al concentrations, respectively. There were 504 and 541 OTUs enriched for BW69 and W270 at the low Al concentration, and 294 and 372 for BW69 and W270 at the high Al concentration, respectively (Figures 6A–D). There were 49 OTUs that overlapped in the enriched OTUs in the rhizosphere of BW69 at both the low and high Al concentrations (Figure 6E and Supplementary Table S2). The effect of different Al concentrations on relative abundance of OTUs (>0.1%) is shown in Figure 7. The relative abundances of OTU15 (*Gammaproteobacteria_KF-JG30-C25_norank*), OTU23 (*Mizugakiibacter*), OTU55 (*Ktedonobacteraceae_uncultured*), OTU65 (*Dyella*), OTU93 (*Alkanibacter*), and OTU195 (*Ktedonobacterales_JG30-KF-AS9_norank*) increased with the increase of Al concentration, with higher values in the rhizosphere of W69compared with W270 under the high Al concentration (Figure 7).

### Co-occurrence Network Analysis of Wild Soybean Rhizosphere Bacteria

**FIGURE 8:**
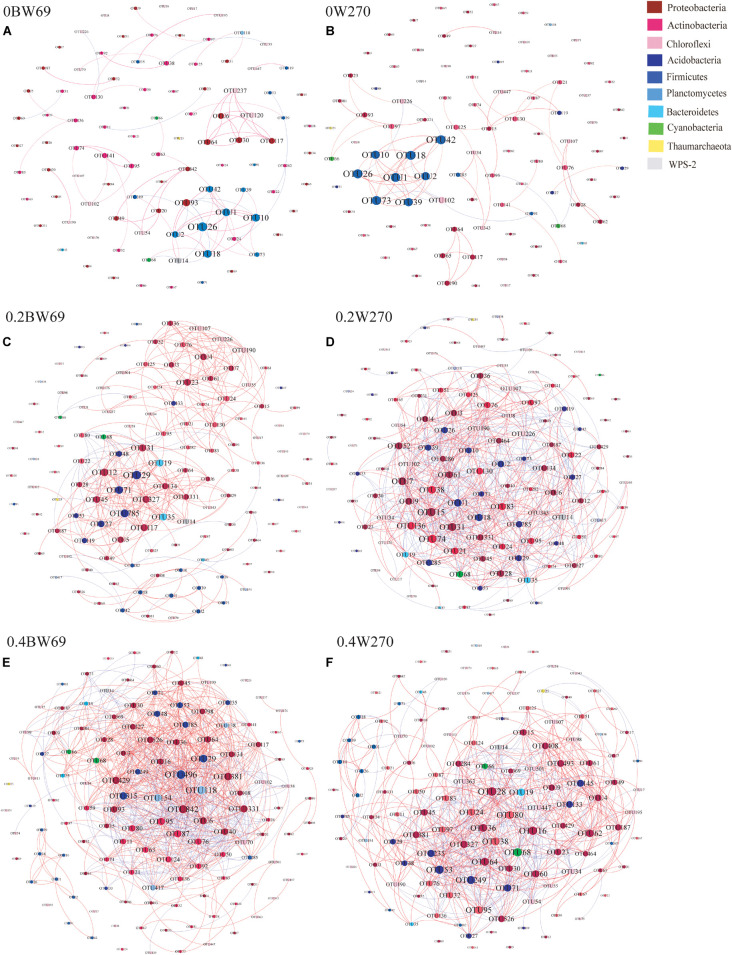
Co-occurrence network of bacterial community in the rhizosphere of genotypes under three Al concentrations, i.e., 0BW69 **(A)**, 0W270 **(B)**, 0.2BW69 **(C)**, 0.2W270 **(D)**, 0.4BW69 **(E)**, and 0.4W270 **(F)**. A connection stands for a strong (Spearman’s *r* > 0.8) and significant (*p* < 0.05) correlation. The co-occurring networks are colored by phyla. For each graph, the size of each node is proportional to the number of connections (degree). A red edge indicates a positive interaction between two individual nodes, whereas a green edge indicates a negative interaction.

To determine the symbiosis and competitive mode of bacterial consortia in the rhizosphere of BW69 and W270, the OTUs with relative abundance >0.1% were screened as nodes (Supplementary Table S3). Six networks were constructed, and the network structure of two genotypes at different Al concentrations was analyzed (Figure 8 and Table 2). The network topology properties, such as the number of edges, positively and negatively correlated edges, and graph density, were similar between BW69 and W270 at the zero Al concentration (Table 2). The number of edges, positively correlated edges, graph density, and average degree in the rhizosphere of W270 were greater than BW69 at the low Al concentration, whereas an opposite tendency was observed at the high Al concentration (Table 2). Keystone bacterial species of the two genotypes at different Al concentrations are presented in Table 3. The OTU496 (*Occallatibacter*) and OTU118 (*Singulisphaera*) were identified as keystone species in the BW69 network at the high Al concentration, whereas OTU16 (*Bradyrhizobium*) and OTU28 (*Pandoraea*) were identified in the rhizosphere of W270 (Table 3).

**TABLE 2:** Topological properties of co-occurring bacterial networks in the rhizosphere of two soybean genotypes under three Al concentrations (0BW69, 0W270, 0.2BW69, 0.2W270, 0.4BW69, and 0.4W270).

Network metrics	0BW69	0W270	0.2BW69	0.2W270	0.4BW69	0.4W270
Number of nodes	98	96	131	131	132	132
Number of edges	182	166	472	792	956	805
Number of positive correlations	166	158	435	476	682	530
Number of negative correlations	16	8	37	316	274	275
Average path length (APL)	1.377	1.954	4.217	2.517	3.296	3.373
Graph density	0.038	0.036	0.055	0.093	0.111	0.093
Network diameter	3	5	12	7	12	11
Average clustering coefficient (*avgCC*)	0.856	0.825	0.723	0.618	0.688	0.645
Average degree (*avgK*)	3.714	3.458	7.206	12.092	14.485	12.197
Interconnecting piece	53	59	36	27	20	19
Modularity (M)	0.922	0.806	0.645	5.95	1.017	2.322

**TABLE 3:** Topological properties of keystone species in the rhizosphere of two soybean genotypes under three Al concentrations (0BW69, 0W270, 0.2BW69, 0.2W270, 0.4BW69, and 0.4W270).

Cultivars	Phylum	Class	Order	Family	Genus	Species	OTU	Degree	Closeness centrality	Betweenness centrality
0BW69	Firmicutes	Bacilli	Lactobacillales	Carno- bacteriaceae	*Carnobacterium*	*Carnobacterium maltaromaticum*	OTU26	12	0.917	14.33
	Firmicutes	Bacilli	Lactobacillales	Streptococcaceae	*Lactococcus*	*Norank*	OTU18	10	0.786	7.83
0W270	Chloroflexi	Ktedono- bacteria	Ktedono- bacterales	Ktedono- bacteraceae	*HSB OF53-F07*	*Uncultured Ktedonobacter* sp.	OTU102	7	0.560	33.30
	Firmicutes	Bacilli	Lactobacillales	Streptococcaceae	*Lactococcus*	*Norank*	OTU1	10	0.609	7.77
0.2BW69	Proteobacteria	Gammaproteo- bacteria	Gammaproteo- bacteria Incertae Sedis	Unknown Family	*Acidibacter*	*Uncultured gammaproteo- bacterium*	OTU31	19	0.349	1099.89
	Acidobacteria	Acidobacteriia	Acido- bacteriales	Acidobacteriaceae (subgroup 1)	*Uncultured*	*Norank*	OTU29	24	0.336	257.58
0.2W270	Firmicutes	Bacilli	Lactobacillales	Strepto- coccaceae	*Lactococcus*	*Norank*	OTU1	29	0.503	292.73
	Proteobacteria	Gammaproteo- bacteria	KF-JG30-C25	Norank	*Norank*	*Uncultured bacterium*	OTU15	34	0.513	148.52
0.4BW69	Acidobacteria	Acidobacteriia	Acido- bacteriales	Acidobacteriaceae (Subgroup 1)	*Occallatibacter*	*Norank*	OTU496	48	0.459	258.65
	Planctomycetes	Planctomycetacia	Isosphaerales	Isosphaeraceae	*Singulisphaera*	*Uncultured bacterium*	OTU118	46	0.439	162.04
0.4W270	Proteobacteria	Alphaproteo- bacteria	Rhizobiales	Xanthobacteraceae	*Bradyrhizobium*	*Norank*	OTU16	31	0.416	331.86
	Proteobacteria	Gammaproteo- bacteria	Betaproteo- bacteriales	Burkholderiaceae	*Pandoraea*	*Pandoraea thiooxydans*	OTU28	32	0.409	222.55

## Discussion

The purpose of this study was to reveal how different levels of Al stress affect bacterial community characteristics in the rhizosphere of wild soybean genotypes differing in the Al tolerance. The results were consistent with our first hypothesis, of which the bacterial community structures in the rhizosphere of BW69 were different from that of W270 at the high Al concentration. There were greater differences in bacterial abundance, Shannon index, and abundances of rhizobacteria at the class, genus, and OTU levels between BW69 and W270 under the high Al concentration than the low Al concentration. However, the second hypothesis was not verified according to our results because no bacteria species that related to Al resistance were recruited in the rhizosphere of BW69.

### Response of Rhizosphere Bacterial Abundance and Diversity to Al Stress

Soil nutrients were positively correlated with bacterial abundance (Sun et al., 2015). In our study, the higher bacterial abundance in the rhizosphere of BW69 compared with W270 might be attributed to more abundant nutrients in the rhizosphere, such as more available phosphorus, NH_4_^+^-N, and NO_3_^–^-N (Table 1). For bacterial diversity, the Shannon index was significantly lower in the rhizosphere of BW69 compared with W270 at the high Al concentration. This finding was inconsistent with our previous research in which the Al-T cultivated soybean genotype had greater bacterial diversity than that of the Al-S soybean genotype (Lian et al., 2019). These inconsistent results may be caused by two reasons. One would be the lower soil pH in the rhizosphere of BW69 compared with W270, posing a negative impact on bacterial diversity that might conceal the positive contribution to more root exudates secreted by the Al-T genotype (Fierer and Jackson, 2006; Rousk et al., 2010; Bartram et al., 2014). Another reason could be associated with the different soybean varieties used in the two studies because the bacterial diversity was significantly different between wild and cultivated crops (Shenton et al., 2016).

### Response of Rhizosphere Bacterial Community Structure to Al Stress

The NMDS showed significant differences in bacterial community structure in the rhizosphere between BW69 and W270 at the high Al stress. This finding was consistent with our previous study which illustrated that high Al stress magnified the differences of bacterial community structure between Al-T and Al-S soybean genotypes (Lian et al., 2019). This may be attributed to the fact that various genotypes have different levels of Al-related gene expression when exposed to Al stress, and these expression levels increased with the increase of Al concentration, especially in the Al-T genotype (Maron et al., 2010; Tovkach et al., 2012). The greater expression of these genes in the Al-T genotypes lead to more secretion of organic acids that could chelate more Al^3+^ to reduce Al toxicity (Yang et al., 2012; Li et al., 2017). For example, the *GsMATE* gene cloned from BW69 was related to the synthesis and secretion of citric acids, which enhanced the tolerance of *Arabidopsis* to Al stress (Ma et al., 2018). However, more secreted organic acids in the Al-T genotype may result in decreased pH, providing a complex environment, and consequently affecting a wide variety of bacteria in the rhizosphere (Table 1) (Clarkson and Marschner, 1995; Jones, 1998; Teplitski et al., 2000; Wang et al., 2013).

We conducted a differential analysis on OTU relative abundance and observed that 49 OTUs were co-enriched in BW69 soybean under the low and high Al concentrations. These OTUs mainly belong to Proteobacteria, Actinobacteria, and Chloroflexi. Some OTUs in BW69 had greater relative abundance than in W270 at the high Al concentration, such as OTU15 (*Gammaproteobacteria_KF-JG30-C25_norank*), OTU23 (*Mizugakiibacter*), OTU55 (*Ktedonobacteraceae_uncultured*), OTU65 (*Dyella*), OTU93 (*Alkanibacter*), and OTU195 (*Ktedonobacterales_JG30-KF-AS9_norank*) (Figure 7). Various functions of these bacteria have been reported. For instance, *Mizugakiibacter* has potential use for the remediation of soil with heavy metal pollution (Guo et al., 2017) and *Alkanibacter* can degrade hexane and other short-chain alkanes (Davis-Belmar et al., 2013). *Ktedonobacteria*, which predominate in acidic soil, may act as a microbial resource with the potential to produce secondary metabolites (Yabe et al., 2017). These bacteria were different from those identified in our previous study in which the co-enriched OTUs of cultivated Al-T soybeans mainly belonged to *Tumebacillus* and *Burkholderia* (Lian et al., 2019). These results indicated that wild and cultivated soybeans recruited different species of bacteria to resist Al stress. However, the relationship between these bacteria and soil Al toxicity needs further study. Whether exogenous addition of these bacteria improves soybean resistance to Al toxicity warrants further test.

The CCA showed that the bacterial community structures of two genotypes significantly correlated with soil properties, except for total potassium. This pattern could be explained by the fact that Al stress had a greater impact on the environmental factors than genotypes, which reflected that some environmental factors such as pH and exchangeable H^+^ and Al^3+^ were significantly different among Al concentrations, without a corresponding difference between two genotypes (Table 1). Moreover, the CCA for the high Al concentration showed that the differences in the bacterial community structure between BW69 and W270 were mainly linked with total nitrogen, NH_4_^+^-N and C:N ratio (Figure 3C). This finding was consistent with our previous study showing that NH_4_^+^-N mainly drove differences in bacterial community structure in the rhizosphere of Al-T and Al-S genotype under high Al concentration (Lian et al., 2019). These results provided more evidence on NH_4_^+^-N mitigating the toxicity of Al to plants (Zhao and Shen, 2018). An increase in NH_4_^+^-N decreases pH, thereby increasing the content of Al^3+^ and H^+^ (Zhao and Shen, 2018). However, Al-T plants may be able to take up more H^+^ and suppress Al^3+^ to enter the plant roots, consequently reducing Al phytotoxicity (Zhao et al., 2009, 2014).

### Response of Bacterial Co-occurrence Networks to Al Stress

A network analysis was constructed to explore the interrelationship among bacteria and compare the structure of the networks in the rhizosphere of BW69 and W270. In this study, the network topology properties, such as the number of edges, positive correlation edges, graph density, and average degree in the rhizosphere of W270, were greater than those of BW69 at the low Al concentration, whereas an opposite trend was observed at the high Al concentration (Figure 8 and Table 2). This may be attributed to the absence of difference in soil pH between two genotypes at the low Al concentration, which might lead to the similar secretion of organic acids between BW69 and W270 (Chen and Liao, 2016; Liu et al., 2016). Combined with the results of network in this study, W270 may even be better adapted to low Al stress. Furthermore, a significant lower value of pH in BW69 than W270 may be caused by the more organic acid expression of BW69 (Liang et al., 2013; Liu et al., 2016; Zhou et al., 2017). More organic acids not only chelate more Al^3+^ but also provide a better living environment (Hu et al., 2018; Lian et al., 2019) for more complicated network structure of BW69 than W270. In addition, the mechanism for recruiting Al-tolerant microorganisms of Al-T and Al-S genotypes were different (Yang et al., 2012; Lian et al., 2019). The Al-tolerant microorganisms recruited by BW69 could cope with different degrees of Al stress and therefore form a more complicated network structure than W270. W270 may stimulate most of the Al-tolerant microorganisms to be involved in the physiological processes associated with the low Al stress. However, many microorganisms in the W270 rhizosphere cannot adapt to high Al stress, and only a small number of Al-tolerant bacteria were recruited in the rhizosphere, which simplified the network structure.

BW69 had lower modularity compared with that of Al-S under Al stress, which could be interpreted as increased inter-species competition, and less resistant to Al stress in the rhizosphere of BW69 than W270 (Saavedra et al., 2011; Fan et al., 2018). Moreover, the keystone species being associated with BW69 and W270, and the difference in keystone species, regardless of their abundance, would be a critical determinant of the other community compositions in the rhizosphere (Zhou et al., 2010; Ma et al., 2016; Jiang et al., 2017a). The difference in network structure between BW69 and W270 under different Al concentrations were inconsistent to our previous study which showed that the Al-S cultivated soybean had a simpler network at the low Al concentration and a more complex network at the high Al concentration compared with Al-T cultivated soybean (Lian et al., 2019). This might be attributed to that wild soybean has stronger resistance to biotic and abiotic stress than cultivated soybean (Dong et al., 2001; Chen et al., 2006; Singh and Hymowitz, 2011). The difference in the root architecture and microhabitat between wild and cultivated soybean could influence the root exudates and the plant immune system, and consequently causing the different bacterial network structure (Shenton et al., 2016). However, more specific reasons of the opposite trend of the network structure between wild and cultivated soybeans should be further studied.

In conclusion, our findings highlighted that high Al concentration magnified the differences of bacterial community structure between two genotypes. Furthermore, 49 OTUs were identified to be co-enriched in the rhizosphere of BW69 at the low and high Al concentrations. Moreover, total nitrogen and NH_4_^+^-N were the major factors affecting the bacterial community structure in the rhizospheres of the two genotypes under the high Al concentration. However, the lower modularity in BW69 under Al stress may cause the rhizobacterial community to be less resistant and more influenced by disturbance. Collectively, this study emphasizes the possibility of using rhizobacteria as an improved crop breeding or gene to produce crops that are more resistant to the toxicity of heavy metal. However, the relationship between these “enriched” bacterial species and soil aluminum toxicity has not been clarified. Further tests should be carried out to evaluate the universality of the results that whether these bacteria can improve the resistance of soybean to aluminum toxicity in different soil types.

## Data Availability Statement

The datasets generated for this study can be found in the NCBI short-read archive under accession number PRJNA531335.

## Author Contributions

TL, HN, YC, and QM designed the research. TL, QS, YL, and ZC performed the research. QS and TL analyzed the data and wrote the manuscript. JJ, RW, and YZ improved the English. All authors contributed to the article and approved the submitted version.

## Conflict of Interest

The authors declare that the research was conducted in the absence of any commercial or financial relationships that could be construed as a potential conflict of interest.
