# Supplementary material for: High Aluminum Drives Different Rhizobacterial Communities Between Aluminum-Tolerant and Aluminum-Sensitive Wild Soybean
Source: Front Microbiol. 2020 Aug 19;11:1996. doi: 10.3389/fmicb.2020.01996 (PMC7466775; doi:10.3389/fmicb.2020.01996)
Supplement: Supplementary file 1 [file Data_Sheet_1.docx]

Table S1 Spearman's correlations between the environmental variables and the bacterial community structure (Bray-Curtis distance) determined by the Mantel test.

| Chemical properties | *P* (BW69) | *r* (BW69) | *P* (W270) | *r* (W270) | *P* (High-Al) | *r* (High-Al) |
| --- | --- | --- | --- | --- | --- | --- |
| TC | **0.001** | 0.498 | **0.001** | 0.561 | 0.240 | 0.132 |
| TN | **0.001** | 0.575 | **0.001** | 0.608 | **0.003** | 0.602 |
| C:N ratio | 0.077 | 0.144 | **0.008** | 0.358 | **0.003** | 0.562 |
| TK | 0.101 | 0.116 | **0.041** | 0.194 | 0.163 | 0.143 |
| TP | **0.003** | 0.342 | **0.001** | 0.723 | 0.256 | 0.068 |
| AP | **0.001** | 0.666 | **0.001** | 0.920 | 0.055 | 0.342 |
| NH_4_^+^-N | **0.012** | 0.337 | **0.001** | 0.818 | **0.003** | 0.594 |
| NO_3_^-^-N | **0.001** | 0.662 | **0.001** | 0.929 | **0.004** | 0.576 |
| Exchangeable H^+^ | **0.001** | 0.805 | **0.001** | 0.812 | 0.901 | -0.198 |
| Exchangeable Al^3+^ | **0.001** | 0.745 | **0.001** | 0.650 | 0.058 | 0.265 |
| pH | **0.001** | 0.924 | **0.001** | 0.963 | **0.009** | 0.625 |

Table S2 Enriched OTUs in the BW69 genotype at both low and high Al concentrations.

| OTUID | **0BW69** | 0W270 | **0.2BW69** | 0.2W270 | **0.4BW69** | 0.4W270 | Phylum | Class | Order | Family | Genus |
| --- | --- | --- | --- | --- | --- | --- | --- | --- | --- | --- | --- |
| OTU15 | 0±0 | 0±0 | 0.04±0.02 | 0.01±0.01 | 2.37±1.14 | 0.11±0.07 | Proteobacteria | Gammaproteobacteria | KF-JG30-C25 | norank | *norank* |
| OTU23 | 0±0 | 0±0 | 0.38±0.14 | 0.41±0.1 | 0.47±0.12 | 0.2±0.07 | Proteobacteria | Gammaproteobacteria | Xanthomonadales | Rhodanobacteraceae | *Mizugakiibacter* |
| OTU55 | 0±0 | 0±0 | 0.03±0.01 | 0.03±0.01 | 0.43±0.1 | 0.25±0.13 | Chloroflexi | Ktedonobacteria | Ktedonobacterales | Ktedonobacteraceae | *uncultured* |
| OTU65 | 0±0 | 0±0 | 0.06±0.05 | 0.06±0.09 | 0.43±0.14 | 0.2±0.28 | Proteobacteria | Gammaproteobacteria | Xanthomonadales | Rhodanobacteraceae | *Dyella* |
| OTU93 | 0±0 | 0±0 | 0.1±0.02 | 0.1±0.05 | 0.29±0.1 | 0.15±0.05 | Proteobacteria | Gammaproteobacteria | Salinisphaerales | Solimonadaceae | *Alkanibacter* |
| OTU195 | 0±0 | 0±0 | 0.04±0.02 | 0.05±0.01 | 0.15±0.02 | 0.11±0.03 | Chloroflexi | Ktedonobacteria | Ktedonobacterales | JG30-KF-AS9 | *norank* |
| OTU6 | 0±0 | 0±0 | 0.6±0.25 | 0.71±0.05 | 1.1±0.24 | 0.91±0.36 | Proteobacteria | Gammaproteobacteria | Gammaproteobactria Incertae Sedis | Unknown Family | *Acidibacter* |
| OTU114 | 0±0 | 0±0 | 0.35±0.1 | 0.38±0.1 | 0.06±0.01 | 0.1±0.02 | Acidobacteria | Subgroup 6 | norank | norank | *norank* |
| OTU84 | 0±0 | 0±0 | 0.27±0.1 | 0.37±0.06 | 0.01±0 | 0.08±0.04 | Bacteroidetes | Bacteroidia | Cytophagales | Microscillaceae | *uncultured* |
| OTU30 | 0±0 | 0±0 | 0.19±0.06 | 0.23±0.06 | 0.77±0.3 | 0.88±0.19 | Proteobacteria | Alphaproteobacteria | Azospirillales | Azospirillaceae | *Skermanella* |
| OTU36 | 0±0 | 0±0 | 0.18±0.03 | 0.23±0.04 | 0.28±0.08 | 0.22±0.08 | Proteobacteria | Gammaproteobacteria | KF-JG30-C25 | norank | *norank* |
| OTU120 | 0±0 | 0±0 | 0.08±0.04 | 0.05±0.01 | 0.21±0.02 | 0.2±0.02 | Chloroflexi | Ktedonobacteria | Ktedonobacterales | JG30-KF-AS9 | *norank* |
| OTU284 | 0±0 | 0±0 | 0.07±0.04 | 0.06±0.01 | 0.11±0.04 | 0.12±0.03 | Proteobacteria | Gammaproteobacteria | Gammaproteobacteria Incertae Sedis | Unknown Family | *Acidibacter* |
| OTU332 | 0±0 | 0±0 | 0.06±0.02 | 0.07±0 | 0.03±0.01 | 0.02±0.01 | Proteobacteria | Alphaproteobacteria | Elsterales | uncultured | *norank* |
| OTU386 | 0±0 | 0±0 | 0.06±0.01 | 0.09±0.02 | 0.06±0.02 | 0.1±0.04 | Planctomycetes | Planctomycetacia | Isosphaerales | Isosphaeraceae | *Singulisphaera* |
| OTU153 | 0±0 | 0±0 | 0.12±0.03 | 0.16±0.04 | 0.05±0.01 | 0.25±0.16 | Proteobacteria | Alphaproteobacteria | Caulobacterales | Caulobacteraceae | *uncultured* |
| OTU416 | 0±0 | 0±0 | 0.05±0.02 | 0.07±0 | 0.05±0.01 | 0.06±0.02 | Planctomycetes | Planctomycetacia | Isosphaerales | Isosphaeraceae | *norank* |
| OTU579 | 0±0 | 0±0 | 0.04±0.03 | 0.02±0.01 | 0.01±0.01 | 0.06±0.02 | Proteobacteria | Gammaproteobacteria | KF-JG30-C25 | norank | *norank* |
| OTU923 | 0±0 | 0±0 | 0.04±0.01 | 0.04±0.02 | 0.02±0.01 | 0.02±0.01 | Actinobacteria | Thermoleophilia | Gaiellales | uncultured | *norank* |
| OTU689 | 0±0 | 0±0 | 0.03±0.01 | 0.07±0.02 | 0.01±0.01 | 0.03±0.02 | Proteobacteria | Alphaproteobacteria | Elsterales | uncultured | *norank* |
| OTU987 | 0±0 | 0±0 | 0.03±0.01 | 0.05±0.02 | 0.02±0 | 0.01±0 | WPS-2 | norank | norank | norank | *norank* |
| OTU53 | 0±0 | 0±0 | 0.14±0.04 | 0.15±0.02 | 0.49±0.11 | 0.71±0.13 | Acidobacteria | Acidobacteriia | Acidobacteriales | Acidobacteriaceae (Subgroup 1) | *uncultured* |
| OTU1861 | 0±0 | 0±0 | 0.02±0.01 | 0.03±0.01 | 0±0 | 0.02±0.01 | Gemmatimonadetes | Gemmatimonadetes | Gemmatimonadales | Gemmatimonadaceae | *uncultured* |
| OTU605 | 0±0 | 0±0 | 0.05±0.02 | 0.06±0.03 | 0.02±0.01 | 0.02±0.01 | Planctomycetes | Planctomycetacia | Planctomycetales | uncultured | *norank* |
| OTU668 | 0±0 | 0±0 | 0.02±0.01 | 0.03±0.02 | 0.05±0.01 | 0.05±0.01 | Chloroflexi | Ktedonobacteria | Ktedonobacterales | JG30-KF-AS9 | *norank* |
| OTU642 | 0±0 | 0±0 | 0.02±0.01 | 0.02±0.01 | 0.05±0.02 | 0.07±0.02 | Chloroflexi | AD3 | norank | norank | *norank* |
| OTU773 | 0±0 | 0±0 | 0.02±0.02 | 0.01±0.01 | 0.06±0.02 | 0.06±0.02 | Acidobacteria | Acidobacteriia | Acidobacteriales | uncultured | *norank* |
| OTU235 | 0±0 | 0±0 | 0.02±0.01 | 0.02±0.01 | 0.19±0.03 | 0.3±0.07 | Acidobacteria | Acidobacteriia | Acidobacteriales | Acidobacteriaceae (Subgroup 1) | *uncultured* |
| OTU922 | 0±0 | 0±0 | 0.02±0.01 | 0.01±0.01 | 0.03±0.02 | 0.02±0.01 | Chloroflexi | Ktedonobacteria | Ktedonobacterales | JG30-KF-AS9 | *norank* |
| OTU530 | 0±0 | 0±0 | 0.02±0.01 | 0.03±0.01 | 0.03±0 | 0.08±0.03 | Chloroflexi | Ktedonobacteria | Ktedonobacterales | JG30-KF-AS9 | *norank* |
| OTU324 | 0±0 | 0±0 | 0.01±0.01 | 0±0 | 0.07±0.02 | 0.01±0.01 | Acidobacteria | Acidobacteriia | Acidobacteriales | Acidobacteriaceae (Subgroup 1) | *uncultured* |
| OTU2320 | 0±0 | 0±0 | 0.01±0.01 | 0.01±0.01 | 0.01±0 | 0.01±0 | WPS-2 | norank | norank | norank | *norank* |
| OTU2824 | 0±0 | 0±0 | 0.01±0.01 | 0.01±0.01 | 0.01±0 | 0±0.01 | Acidobacteria | Acidobacteriia | Acidobacteriales | Koribacteraceae | *Candidatus Koribacter* |
| OTU5480 | 0±0 | 0.01±0.01 | 0±0 | 0±0 | 0±0 | 0±0 | Proteobacteria | Deltaproteobacteria | NB1-j | norank | *norank* |
| OTU1253 | 0±0 | 0.01±0.01 | 0.03±0.02 | 0.02±0.01 | 0.03±0.01 | 0.02±0.01 | Chloroflexi | TK10 | norank | norank | *norank* |
| OTU1207 | 0±0 | 0±0 | 0.01±0.01 | 0.01±0.01 | 0.01±0.01 | 0.01±0.01 | Proteobacteria | Gammaproteobacteria | Legionellales | Legionellaceae | *Legionella* |
| OTU1244 | 0±0 | 0.01±0 | 0.01±0.01 | 0.03±0.03 | 0.02±0.01 | 0.02±0.02 | Actinobacteria | Actinobacteria | Catenulisporales | Catenulisporaceae | *Catenulispora* |
| OTU713 | 0±0 | 0.01±0 | 0.02±0.01 | 0.02±0.02 | 0.02±0.01 | 0.02±0.01 | Chloroflexi | Chloroflexia | Thermomicrobiales | JG30-KF-CM45 | *norank* |
| OTU1010 | 0.01±0 | 0.01±0.01 | 0.05±0.01 | 0.02±0.01 | 0.02±0.01 | 0.02±0.01 | Actinobacteria | Thermoleophilia | Gaiellales | uncultured | *norank* |
| OTU1640 | 0±0 | 0±0 | 0.01±0.01 | 0.02±0 | 0.01±0.01 | 0.02±0.01 | Planctomycetes | Planctomycetacia | Isosphaerales | Isosphaeraceae | *Singulisphaera* |
| OTU691 | 0.01±0.01 | 0.02±0 | 0.03±0.02 | 0.02±0.01 | 0.02±0 | 0.02±0.01 | Actinobacteria | Thermoleophilia | Gaiellales | uncultured | *norank* |
| OTU1285 | 0±0.01 | 0.01±0 | 0.01±0 | 0.01±0.01 | 0.02±0.01 | 0.03±0.02 | Acidobacteria | Acidobacteriia | Acidobacteriales | uncultured | *norank* |
| OTU696 | 0.01±0.01 | 0.02±0.01 | 0.03±0.01 | 0.03±0 | 0.03±0.01 | 0.02±0.01 | Actinobacteria | Thermoleophilia | Gaiellales | uncultured | *norank* |
| OTU1189 | 0.01±0 | 0.01±0.01 | 0.03±0.01 | 0.01±0.01 | 0.02±0.01 | 0.02±0.01 | Actinobacteria | Actinobacteria | Propionibacteriales | Propionibacteriaceae | *Microlunatus* |
| OTU1422 | 0.01±0 | 0.01±0.01 | 0.02±0.01 | 0.01±0.01 | 0.01±0 | 0.01±0 | Actinobacteria | Thermoleophilia | Gaiellales | uncultured | *norank* |
| OTU986 | 0±0 | 0±0 | 0.01±0 | 0.06±0.12 | 0.03±0.01 | 0.01±0.01 | Acidobacteria | Acidobacteriia | Solibacterales | Solibacteraceae  (Subgroup 3) | *Bryobacter* |
| OTU1219 | 0.01±0.01 | 0.01±0.01 | 0.01±0 | 0.01±0.01 | 0.02±0.01 | 0.02±0.01 | Actinobacteria | Actinobacteria | Micrococcales | Intrasporangiaceae | *Intrasporangium* |
| OTU236 | 0.03±0.01 | 0.05±0.02 | 0.06±0.02 | 0.05±0.02 | 0.07±0.01 | 0.06±0.01 | Actinobacteria | Actinobacteria | Propionibacteriales | Nocardioidaceae | *Nocardioides* |
| OTU136 | 0.07±0.02 | 0.07±0.03 | 0.12±0.03 | 0.09±0.02 | 0.11±0.03 | 0.08±0.04 | Actinobacteria | Thermoleophilia | Gaiellales | Gaiellaceae | *Gaiella* |

Table S3 OTUs with relative abundance greater than 0.1%.

| OTU ID | Phylum | Class | Order | Family | Genus | Species |
| --- | --- | --- | --- | --- | --- | --- |
| OTU3 | Proteobacteria | Gammaproteobacteria | KF-JG30-C25 | norank | *norank* | uncultured gamma proteobacterium |
| OTU4 | Proteobacteria | Gammaproteobacteria | Xanthomonadales | Rhodanobacteraceae | *Rhodanobacter* | uncultured gamma proteobacterium |
| OTU2 | Firmicutes | Bacilli | Bacillales | Bacillaceae | *Bacillus* | Bacillus anthracis |
| OTU1 | Firmicutes | Bacilli | Lactobacillales | Streptococcaceae | *Lactococcus* | norank |
| OTU7 | Proteobacteria | Gammaproteobacteria | Xanthomonadales | Rhodanobacteraceae | *Rhodanobacter* | uncultured gamma proteobacterium |
| OTU15 | Proteobacteria | Gammaproteobacteria | KF-JG30-C25 | norank | *norank* | uncultured bacterium |
| OTU1331 | Proteobacteria | Gammaproteobacteria | Gammaproteobacteria Incertae Sedis | Unknown Family | *Acidibacter* | uncultured gamma proteobacterium |
| OTU5 | Proteobacteria | Gammaproteobacteria | Xanthomonadales | Rhodanobacteraceae | *Rhodanobacter* | uncultured bacterium |
| OTU8 | Chloroflexi | Ktedonobacteria | Ktedonobacterales | JG30-KF-AS9 | *norank* | uncultured bacterium |
| OTU9 | Proteobacteria | Gammaproteobacteria | Betaproteobacteriales | Burkholderiaceae | *Burkholderia-Caballeronia-Paraburkholderia* | norank |
| OTU6 | Proteobacteria | Gammaproteobacteria | Gammaproteobacteria Incertae Sedis | Unknown Family | *Acidibacter* | uncultured gamma proteobacterium |
| OTU31 | Proteobacteria | Gammaproteobacteria | Gammaproteobacteria Incertae Sedis | Unknown Family | *Acidibacter* | uncultured gamma proteobacterium |
| OTU59 | Actinobacteria | Actinobacteria | Catenulisporales | Actinospicaceae | *Actinospica* | norank |
| OTU48 | Acidobacteria | Acidobacteriia | Acidobacteriales | Acidobacteriaceae (Subgroup 1) | *uncultured* | uncultured bacterium |
| OTU29 | Acidobacteria | Acidobacteriia | Acidobacteriales | Acidobacteriaceae (Subgroup 1) | *uncultured* | norank |
| OTU30 | Proteobacteria | Alphaproteobacteria | Azospirillales | Azospirillaceae | *Skermanella* | uncultured bacterium |
| OTU61 | Proteobacteria | Gammaproteobacteria | KF-JG30-C25 | norank | *norank* | uncultured bacterium |
| OTU28 | Proteobacteria | Gammaproteobacteria | Betaproteobacteriales | Burkholderiaceae | *Pandoraea* | Pandoraea thiooxydans |
| OTU20 | Proteobacteria | Gammaproteobacteria | Xanthomonadales | Rhodanobacteraceae | *Rhodanobacter* | norank |
| OTU22 | Actinobacteria | Actinobacteria | Catenulisporales | Actinospicaceae | *Actinospica* | norank |
| OTU12 | Proteobacteria | Alphaproteobacteria | Elsterales | uncultured | *norank* | uncultured alpha proteobacterium |
| OTU24 | Actinobacteria | Thermoleophilia | Gaiellales | uncultured | *norank* | uncultured bacterium |
| OTU14 | WPS-2 | norank | norank | norank | *norank* | uncultured bacterium |
| OTU17 | Chloroflexi | Ktedonobacteria | Ktedonobacterales | JG30-KF-AS9 | *norank* | norank |
| OTU52 | Proteobacteria | Gammaproteobacteria | Xanthomonadales | Rhodanobacteraceae | *Chujaibacter* | norank |
| OTU40 | Proteobacteria | Gammaproteobacteria | Gammaproteobacteria Incertae Sedis | Unknown Family | *Acidibacter* | norank |
| OTU10 | Firmicutes | Bacilli | Lactobacillales | Streptococcaceae | *Lactococcus* | norank |
| OTU11 | Actinobacteria | Actinobacteria | Micrococcales | Intrasporangiaceae | *Oryzihumus* | Oryzihumus leptocrescens |
| OTU49 | Proteobacteria | Gammaproteobacteria | Gammaproteobacteria Incertae Sedis | Unknown Family | *Acidibacter* | uncultured gamma proteobacterium |
| OTU64 | Proteobacteria | Alphaproteobacteria | Acetobacterales | Acetobacteraceae | *uncultured* | uncultured soil bacterium |
| OTU53 | Acidobacteria | Acidobacteriia | Acidobacteriales | Acidobacteriaceae (Subgroup 1) | *uncultured* | uncultured bacterium |
| OTU23 | Proteobacteria | Gammaproteobacteria | Xanthomonadales | Rhodanobacteraceae | *Mizugakiibacter* | uncultured gamma proteobacterium |
| OTU35 | Bacteroidetes | Ignavibacteria | OPB56 | norank | *norank* | uncultured bacterium |
| OTU19 | Bacteroidetes | Bacteroidia | Sphingobacteriales | Sphingobacteriaceae | *Mucilaginibacter* | norank |
| OTU65 | Proteobacteria | Gammaproteobacteria | Xanthomonadales | Rhodanobacteraceae | *Dyella* | norank |
| OTU55 | Chloroflexi | Ktedonobacteria | Ktedonobacterales | Ktedonobacteraceae | *uncultured* | uncultured bacterium |
| OTU67 | Actinobacteria | Actinobacteria | Catenulisporales | Actinospicaceae | *Actinospica* | norank |
| OTU16 | Proteobacteria | Alphaproteobacteria | Rhizobiales | Xanthobacteraceae | *Bradyrhizobium* | norank |
| OTU32 | Actinobacteria | Actinobacteria | Frankiales | Acidothermaceae | *Acidothermus* | uncultured bacterium |
| OTU62 | Proteobacteria | Gammaproteobacteria | Gammaproteobacteria Incertae Sedis | Unknown Family | *Acidibacter* | uncultured bacterium |
| OTU71 | Acidobacteria | Acidobacteriia | Acidobacteriales | Acidobacteriaceae (Subgroup 1) | *uncultured* | uncultured bacterium |
| OTU43 | Bacteroidetes | Bacteroidia | Chitinophagales | Chitinophagaceae | *uncultured* | metagenome |
| OTU26 | Firmicutes | Bacilli | Lactobacillales | Carnobacteriaceae | *Carnobacterium* | Carnobacterium maltaromaticum |
| OTU118 | Planctomycetes | Planctomycetacia | Isosphaerales | Isosphaeraceae | *Singulisphaera* | uncultured bacterium |
| OTU21 | Actinobacteria | Thermoleophilia | Gaiellales | uncultured | *norank* | uncultured bacterium |
| OTU145 | Acidobacteria | Acidobacteriia | Acidobacteriales | Acidobacteriaceae (Subgroup 1) | *Acidipila* | uncultured bacterium |
| OTU117 | Proteobacteria | Deltaproteobacteria | Oligoflexales | 0319-6G20 | *norank* | uncultured bacterium |
| OTU18 | Firmicutes | Bacilli | Lactobacillales | Streptococcaceae | *Lactococcus* | norank |
| OTU133 | Acidobacteria | Acidobacteriia | Acidobacteriales | Acidobacteriaceae (Subgroup 1) | *uncultured* | norank |
| OTU93 | Proteobacteria | Gammaproteobacteria | Salinisphaerales | Solimonadaceae | *Alkanibacter* | uncultured bacterium |
| OTU38 | Actinobacteria | Thermoleophilia | Gaiellales | uncultured | *norank* | uncultured bacterium |
| OTU36 | Proteobacteria | Gammaproteobacteria | KF-JG30-C25 | norank | *norank* | uncultured gamma proteobacterium |
| OTU68 | Cyanobacteria | Oxyphotobacteria | Chloroplast | norank | *norank* | norank |
| OTU369 | Proteobacteria | Gammaproteobacteria | KF-JG30-C25 | norank | *norank* | uncultured gamma proteobacterium |
| OTU45 | Proteobacteria | Alphaproteobacteria | Acetobacterales | Acetobacteraceae | *norank* | norank |
| OTU60 | Proteobacteria | Deltaproteobacteria | Myxococcales | Polyangiaceae | *Pajaroellobacter* | uncultured bacterium |
| OTU54 | Chloroflexi | Ktedonobacteria | Ktedonobacterales | JG30-KF-AS9 | *norank* | uncultured bacterium |
| OTU315 | Acidobacteria | Acidobacteriia | Acidobacteriales | Acidobacteriaceae (Subgroup 1) | *Acidipila* | uncultured bacterium |
| OTU154 | Planctomycetes | Planctomycetacia | Pirellulales | Pirellulaceae | *uncultured* | norank |
| OTU121 | Actinobacteria | Actinobacteria | Catenulisporales | Actinospicaceae | *Actinospica* | norank |
| OTU51 | Actinobacteria | Thermoleophilia | Gaiellales | uncultured | *norank* | uncultured bacterium |
| OTU27 | Acidobacteria | Acidobacteriia | Acidobacteriales | uncultured | *norank* | uncultured bacterium |
| OTU107 | Chloroflexi | Ktedonobacteria | Ktedonobacterales | JG30-KF-AS9 | *norank* | uncultured bacterium |
| OTU75 | Chloroflexi | SHA-26 | norank | norank | *norank* | uncultured bacterium |
| OTU429 | Proteobacteria | Alphaproteobacteria | Acetobacterales | Acetobacteraceae | *uncultured* | uncultured bacterium |
| OTU39 | Firmicutes | Bacilli | Lactobacillales | Streptococcaceae | *Streptococcus* | norank |
| OTU58 | Chloroflexi | Ktedonobacteria | Ktedonobacterales | JG30-KF-AS9 | *norank* | uncultured bacterium |
| OTU190 | Chloroflexi | Ktedonobacteria | Ktedonobacterales | JG30-KF-AS9 | *norank* | uncultured bacterium |
| OTU120 | Chloroflexi | Ktedonobacteria | Ktedonobacterales | JG30-KF-AS9 | *norank* | norank |
| OTU138 | Planctomycetes | Planctomycetacia | Pirellulales | Pirellulaceae | *uncultured* | uncultured bacterium |
| OTU42 | Firmicutes | Bacilli | Lactobacillales | Enterococcaceae | *Enterococcus* | norank |
| OTU363 | Chloroflexi | Ktedonobacteria | Ktedonobacterales | Ktedonobacteraceae | *uncultured* | uncultured bacterium |
| OTU496 | Acidobacteria | Acidobacteriia | Acidobacteriales | Acidobacteriaceae (Subgroup 1) | *Occallatibacter* | norank |
| OTU447 | Chloroflexi | Ktedonobacteria | Ktedonobacterales | Ktedonobacteraceae | *uncultured* | uncultured bacterium |
| OTU97 | Actinobacteria | Thermoleophilia | Gaiellales | uncultured | *norank* | uncultured bacterium |
| OTU34 | Chloroflexi | SHA-26 | norank | norank | *norank* | uncultured bacterium |
| OTU235 | Acidobacteria | Acidobacteriia | Acidobacteriales | Acidobacteriaceae (Subgroup 1) | *uncultured* | uncultured bacterium |
| OTU285 | Firmicutes | Clostridia | Clostridiales | Family XVII | *uncultured* | uncultured bacterium |
| OTU501 | Chloroflexi | Ktedonobacteria | Ktedonobacterales | Ktedonobacteraceae | *uncultured* | uncultured bacterium |
| OTU381 | Proteobacteria | Gammaproteobacteria | Gammaproteobacteria Incertae Sedis | Unknown Family | *Acidibacter* | uncultured bacterium |
| OTU76 | Actinobacteria | Thermoleophilia | Gaiellales | uncultured | *norank* | uncultured bacterium |
| OTU286 | Proteobacteria | Gammaproteobacteria | Xanthomonadales | Rhodanobacteraceae | *Chujaibacter* | norank |
| OTU50 | Actinobacteria | Actinobacteria | Propionibacteriales | Nocardioidaceae | *Marmoricola* | norank |
| OTU74 | Actinobacteria | Thermoleophilia | Gaiellales | uncultured | *norank* | norank |
| OTU195 | Chloroflexi | Ktedonobacteria | Ktedonobacterales | JG30-KF-AS9 | *norank* | uncultured bacterium |
| OTU526 | Proteobacteria | Alphaproteobacteria | Acetobacterales | Acetobacteraceae | *uncultured* | uncultured soil bacterium |
| OTU282 | Actinobacteria | Thermoleophilia | Gaiellales | uncultured | *norank* | uncultured bacterium |
| OTU113 | Chloroflexi | Ktedonobacteria | Ktedonobacterales | JG30-KF-AS9 | *norank* | uncultured bacterium |
| OTU798 | Proteobacteria | Gammaproteobacteria | Steroidobacterales | Woeseiaceae | *Woeseia* | uncultured gamma proteobacterium |
| OTU125 | Actinobacteria | Actinobacteria | Frankiales | Frankiaceae | *Jatrophihabitans* | uncultured bacterium |
| OTU167 | Actinobacteria | Thermoleophilia | Gaiellales | uncultured | *norank* | uncultured bacterium |
| OTU417 | Planctomycetes | Planctomycetacia | Isosphaerales | Isosphaeraceae | *Singulisphaera* | uncultured Singulisphaera sp. |
| OTU134 | Proteobacteria | Gammaproteobacteria | Gammaproteobacteria Incertae Sedis | Unknown Family | *Acidibacter* | uncultured gamma proteobacterium |
| OTU92 | Actinobacteria | Actinobacteria | Frankiales | Acidothermaceae | *Acidothermus* | norank |
| OTU325 | Actinobacteria | Thermoleophilia | Solirubrobacterales | Solirubrobacteraceae | *Conexibacter* | uncultured bacterium |
| OTU98 | Chloroflexi | Ktedonobacteria | Ktedonobacterales | JG30-KF-AS9 | *norank* | uncultured bacterium |
| OTU69 | Proteobacteria | Gammaproteobacteria | Xanthomonadales | Rhodanobacteraceae | *Dyella* | uncultured Dyella sp. |
| OTU130 | Actinobacteria | Thermoleophilia | Gaiellales | uncultured | *norank* | uncultured bacterium |
| OTU170 | Chloroflexi | Anaerolineae | RBG-13-54-9 | norank | *norank* | uncultured bacterium |
| OTU119 | Acidobacteria | Acidobacteriia | Acidobacteriales | Acidobacteriaceae (Subgroup 1) | *Acidipila* | uncultured Acidobacteria bacterium |
| OTU408 | Proteobacteria | Gammaproteobacteria | Xanthomonadales | Rhodanobacteraceae | *Dyella* | uncultured Dyella sp. |
| OTU249 | Acidobacteria | Acidobacteriia | Acidobacteriales | Acidobacteriaceae (Subgroup 1) | *Occallatibacter* | norank |
| OTU102 | Chloroflexi | Ktedonobacteria | Ktedonobacterales | Ktedonobacteraceae | *HSB OF53-F07* | uncultured Ktedonobacter sp. |
| OTU95 | Actinobacteria | Actinobacteria | Corynebacteriales | Mycobacteriaceae | *Mycobacterium* | uncultured bacterium |
| OTU493 | Proteobacteria | Gammaproteobacteria | Xanthomonadales | Rhodanobacteraceae | *Fulvimonas* | norank |
| OTU73 | Firmicutes | Bacilli | Lactobacillales | Leuconostocaceae | *Leuconostoc* | norank |
| OTU228 | Actinobacteria | Actinobacteria | Micrococcales | Intrasporangiaceae | *Janibacter* | norank |
| OTU182 | Firmicutes | Clostridia | Clostridiales | Family XVII | *uncultured* | uncultured bacterium |
| OTU63 | Actinobacteria | Thermoleophilia | Gaiellales | Gaiellaceae | *Gaiella* | uncultured bacterium |
| OTU136 | Actinobacteria | Thermoleophilia | Gaiellales | Gaiellaceae | *Gaiella* | norank |
| OTU83 | Actinobacteria | Thermoleophilia | Solirubrobacterales | 67-14 | *norank* | uncultured bacterium |
| OTU231 | Proteobacteria | Alphaproteobacteria | Sphingomonadales | Sphingomonadaceae | *Sphingomonas* | norank |
| OTU327 | Proteobacteria | Alphaproteobacteria | Micropepsales | Micropepsaceae | *uncultured* | norank |
| OTU91 | Firmicutes | Bacilli | Bacillales | Bacillaceae | *Oceanobacillus* | norank |
| OTU124 | Actinobacteria | Actinobacteria | Propionibacteriales | Nocardioidaceae | *Nocardioides* | norank |
| OTU176 | Chloroflexi | Ktedonobacteria | Ktedonobacterales | JG30-KF-AS9 | *norank* | uncultured bacterium |
| OTU25 | Thaumarchaeota | Nitrososphaeria | Nitrososphaerales | Nitrososphaeraceae | *norank* | uncultured bacterium |
| OTU226 | Chloroflexi | Ktedonobacteria | Ktedonobacterales | JG30-KF-AS9 | *norank* | uncultured bacterium |
| OTU141 | Actinobacteria | Thermoleophilia | Gaiellales | uncultured | *norank* | uncultured bacterium |
| OTU70 | Chloroflexi | Ktedonobacteria | Ktedonobacterales | Ktedonobacteraceae | *HSB OF53-F07* | uncultured Ktedonobacter sp. |
| OTU284 | Proteobacteria | Gammaproteobacteria | Gammaproteobacteria Incertae Sedis | Unknown Family | *Acidibacter* | norank |
| OTU165 | Actinobacteria | Thermoleophilia | Gaiellales | uncultured | *norank* | uncultured bacterium |
| OTU80 | Actinobacteria | Actinobacteria | Streptomycetales | Streptomycetaceae | *Streptomyces* | norank |
| OTU187 | Proteobacteria | Alphaproteobacteria | Elsterales | uncultured | *norank* | uncultured bacterium |
| OTU87 | Actinobacteria | Actinobacteria | Propionibacteriales | Nocardioidaceae | *Nocardioides* | uncultured bacterium |
| OTU785 | Acidobacteria | Acidobacteriia | Acidobacteriales | Acidobacteriaceae (Subgroup 1) | *uncultured* | norank |
| OTU237 | Chloroflexi | Ktedonobacteria | Ktedonobacterales | JG30-KF-AS9 | *norank* | uncultured bacterium |
| OTU66 | Cyanobacteria | Oxyphotobacteria | Chloroplast | norank | *norank* | norank |
| OTU842 | Proteobacteria | Alphaproteobacteria | Acetobacterales | Acetobacteraceae | *uncultured* | uncultured soil bacterium |
| OTU343 | Chloroflexi | Ktedonobacteria | Ktedonobacterales | JG30-KF-AS9 | *norank* | uncultured bacterium |
| OTU464 | Proteobacteria | Gammaproteobacteria | Xanthomonadales | Rhodanobacteraceae | *Chujaibacter* | norank |
| OTU108 | Proteobacteria | Gammaproteobacteria | Gammaproteobacteria Incertae Sedis | Unknown Family | *Acidibacter* | norank |


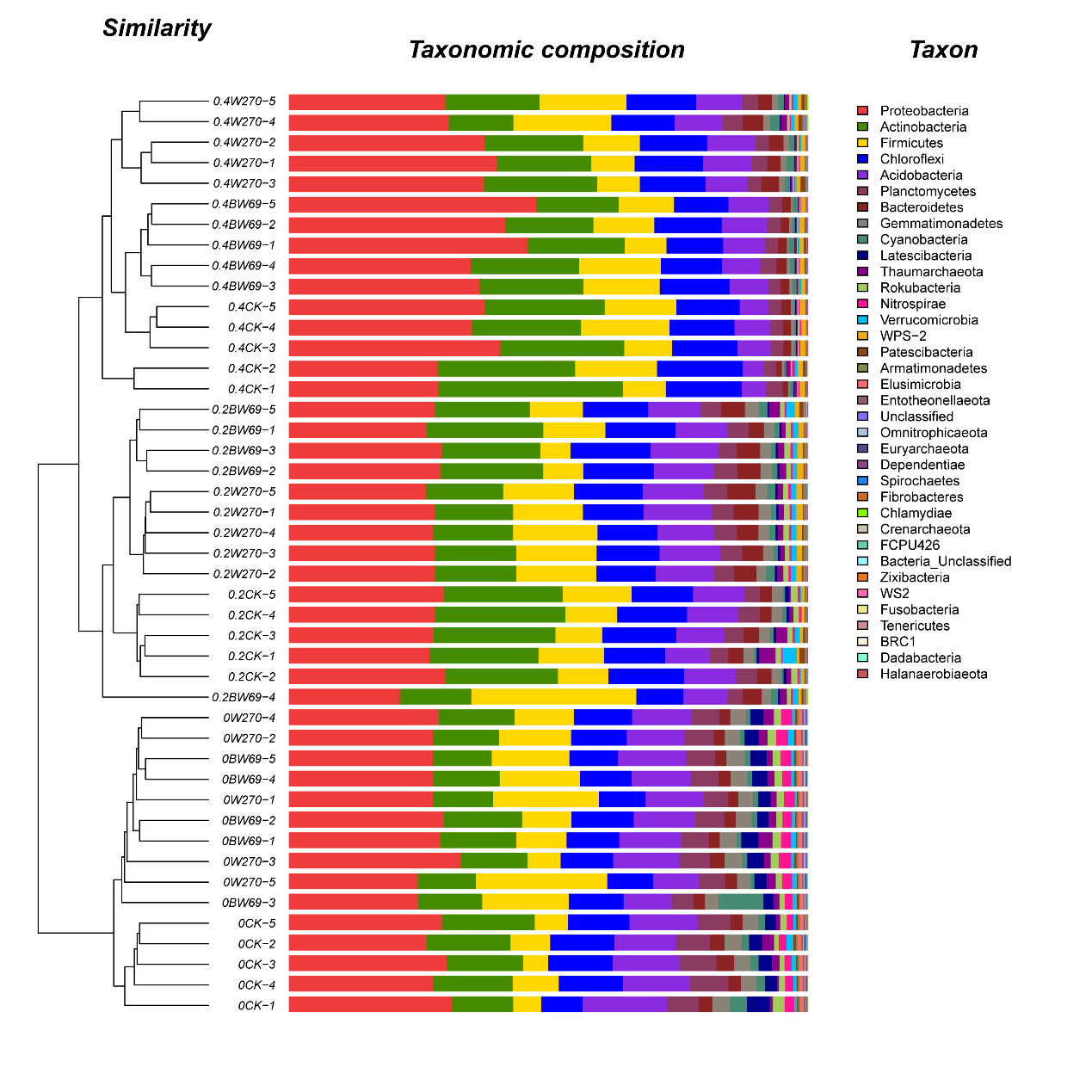


Fig. S1. Phylogenetic relationships of bacterial communities shown together with the relative abundances of different bacterial phyla.
